# Case report: Tenecteplase for acute ischemic stroke after heparin reversal

**DOI:** 10.3389/fstro.2024.1375473

**Published:** 2024-04-23

**Authors:** Manali Desai, Ameen Fahad, Kristi Anderson, Michael Erdman, Scott Silliman

**Affiliations:** College of Medicine—Jacksonville, University of Florida, Jacksonville, FL, United States

**Keywords:** tenecteplase, heparin, protamine sulfate, acute ischemic stroke, TNKase

## Abstract

Intravenous thrombolysis can be administered to appropriate patients with suspected acute ischemic strokes who are on intravenous heparin infusions after its rapid reversal with protamine sulfate. Several case reports suggest the safety of tissue-type plasminogen activator, or alteplase, in these scenarios. Noting the increasing preferential use of tenecteplase over alteplase for intravenous thrombolysis of acute ischemic stroke, the safe and efficacious use of tenecteplase following heparin reversal has not been demonstrated in the literature. Our case demonstrates successful use of intravenous tenecteplase in a patient who was anticoagulated with therapeutic heparin. The patient had no hemorrhagic complications and had an excellent neurological outcome.

## Introduction

Endogenous tissue-type plasminogen activator (tPA) is released from endothelial cells and catalyzes the cleavage of plasminogen to plasmin resulting in degradation of fibrin in thrombi. Synthesis of a wild-type, recombinant tPA called alteplase has allowed for the therapeutic fibrinolysis of thrombi in the setting of myocardial infarctions, pulmonary emboli, and ischemic strokes (Warach et al., 2020). Tenecteplase is a more recently developed tissue-type plasminogen activator that has gained popularity given its advantageous drug characteristics. Compared to alteplase, tenecteplase has a longer half-life and greater fibrin specificity which reduces fibrin depletion. These factors allow for bolus administration and reduction in the risk of systemic bleeding (Panezai et al., 2021).

Thrombolysis is sometimes limited by several exclusionary criteria that make acute ischemic stroke (AIS) patients ineligible for treatment. One of these criteria is the recent use of intravenous heparin and an activated prolonged partial thromboplastin time (PTT). In an effort to allow patients access to treatment despite these limitations, several case reports and case series have evaluated the use of alteplase in this setting. These reports have demonstrated that thrombolysis with alteplase after reversal of heparin with protamine sulfate can be safe. However, to our knowledge, there are no published cases that have documented the use of intravenous thrombolysis with tenecteplase for AIS following heparin reversal. We present a successful case of thrombolysis with tenecteplase after the reversal of heparin with protamine sulfate.

## Case

**Figure 1:**
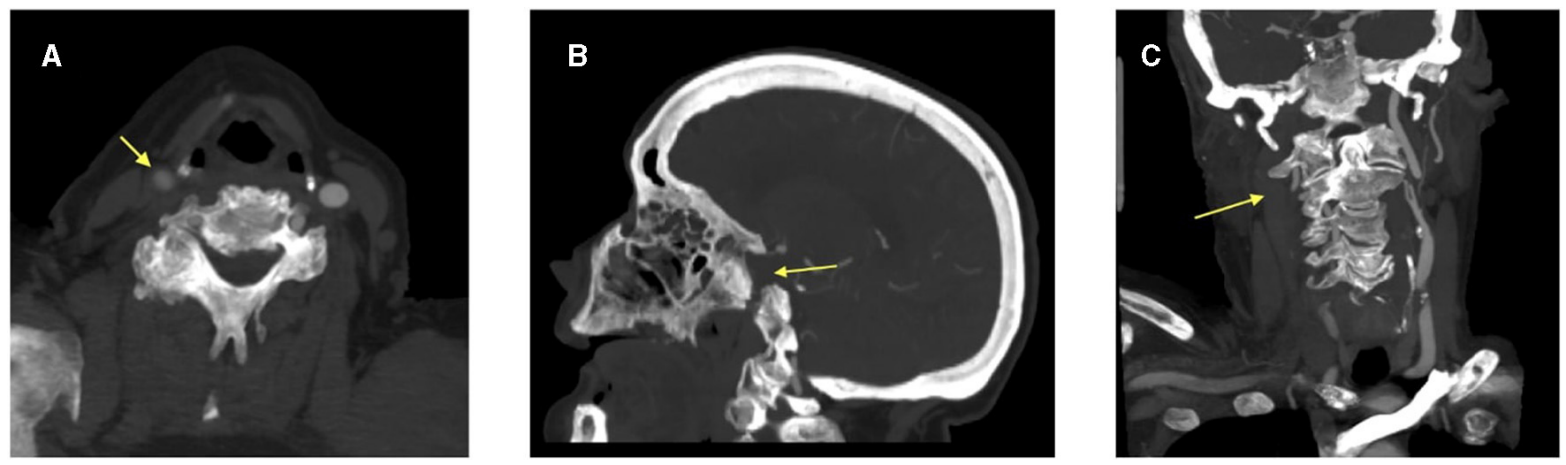
CT angiogram images demonstrating **(A)** reduced contrast opacification of the right common carotid artery on axial view and absent right internal carotid artery on both **(B)** sagittal and **(C)** coronal views. The arrow head is pointing to the right common carotid artery and the absent right internal carotid artery.

**Figure 2:**
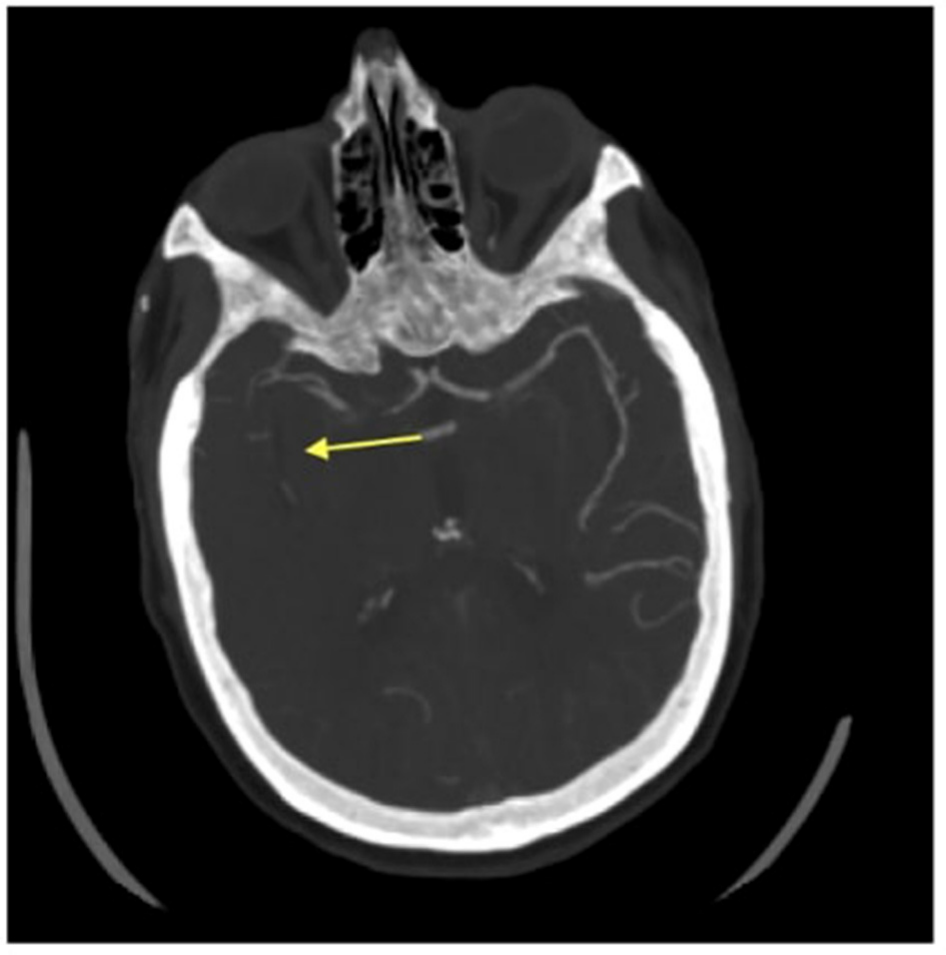
Axial CT angiogram image of an absent inferior division of the M2 segment of the right middle cerebral artery. The arrow head is pointing to where there is an absent right middle cerebral artery.

A 76-year-old Caucasian woman with a past medical history of a myocardial infarction status post percutaneous coronary intervention, atrial fibrillation, hyperlipidemia, hypertension, and prior ischemic stroke presented to a comprehensive stroke center with chest pain. She was found to be in atrial fibrillation with a rapid ventricular response requiring admission to the cardiac intensive care unit and initiation of a therapeutic heparin drip. Shortly after the admission, the patient developed sudden onset left hemiparesis, right gaze preference, and dysarthria, concerning for a right middle cerebral artery (MCA) infarction syndrome. Her last seen normal was ~45 min prior to detection of her deficit. Her NIH stroke score was 12. Prior to transport to the CT scanner, her heparin drip was discontinued. CT head without contrast demonstrated a hyperdensity within the right inferior M2 division of the middle cerebral artery. CT angiogram of her head and neck showed an extensively occluded right internal carotid artery (ICA) from the origin that extended intracranially (Figure 1). There was minimal reconstitution of flow in the distal supraclinoid segment. The M1 segment of the right middle cerebral artery was patent, but an abrupt occlusion was present in the inferior M2 division (Figure 2).

Since the patient was within the time window for thrombolysis, a decision was made to undergo heparin reversal with protamine sulfate and proceed with administration of tenecteplase. 50 mg of protamine sulfate was administered intravenously via a slow push. Following a 10-min wait after receipt of the protamine sulfate, she was treated with 0.25 mg/kg tenecteplase via intravenous push. Within 20 min following tenecteplase administration, the patient was noted to have marked improvement in her symptoms with only a residual mild left hemiparesis and dysarthria. The patient's case was discussed with the neuro-interventional team, who deemed that intervention via mechanical thrombectomy was technically complicated as her right ICA extensive occlusion would make access to the right MCA thrombus difficult to retrieve. Additionally, the patient had rapid symptomatic improvement following thrombolysis, making the risks of intervention greater than its potential benefit.

**Figure 3:**
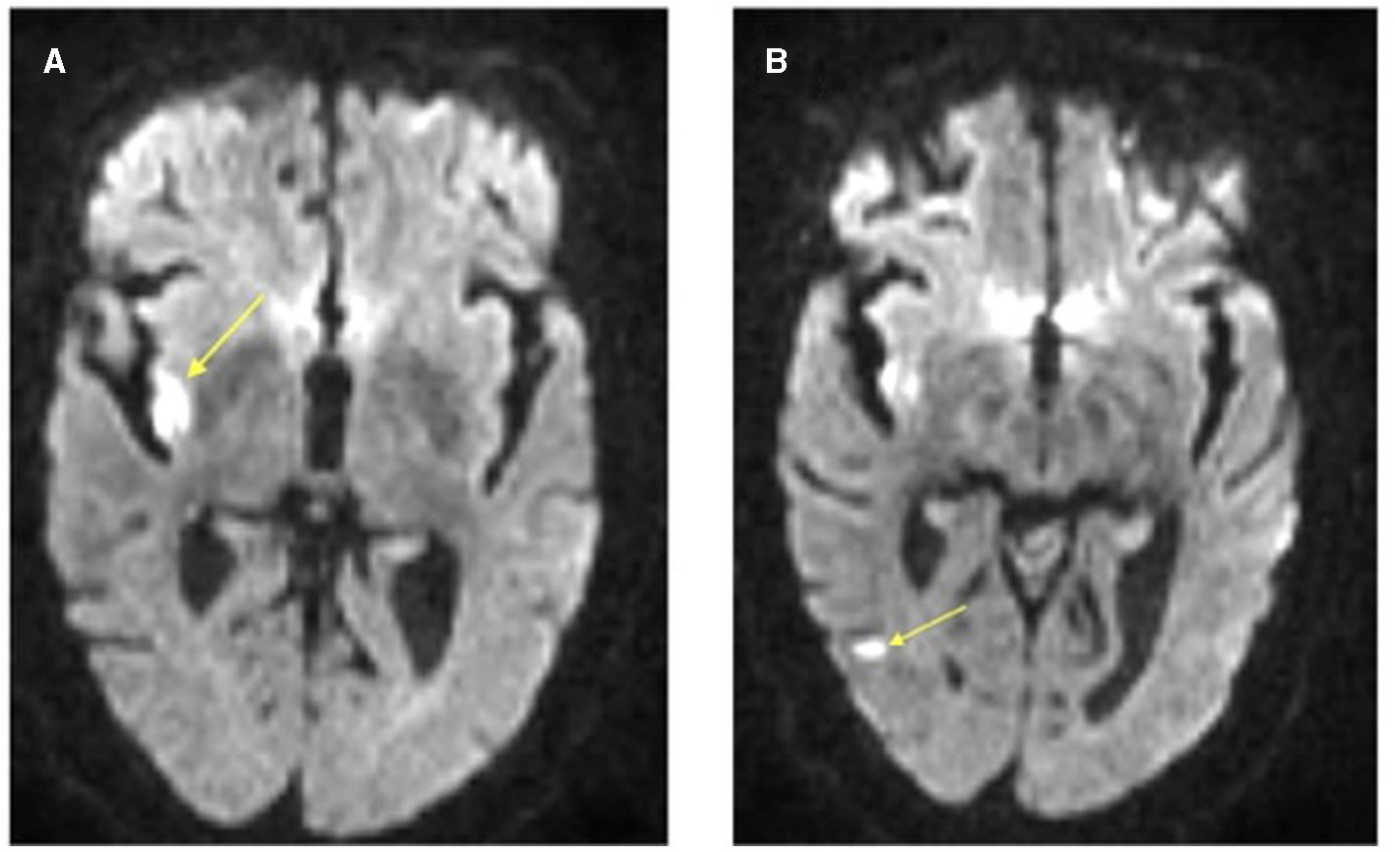
Axial brain MRI DWI sequence illustrating acute infarcts within the **(A)** right posterior insula and **(B)** right posterior temporal lobe. The arrow head is pointing to the areas of restricted diffusion on MRI indicating regions of acute infarct.

A repeat CT Head without contrast was obtained ~24 h after tenecteplase administration without evidence of hemorrhagic transformation and no new infarct. Brain MRI without contrast was completed around the 24-h mark as well and demonstrated several small areas of acute infarct within the right middle cerebral artery distribution, most prominently within the posterior right insula. There were also additional small areas of acute infarct within the posterior right temporal lobe and the right frontal lobe (Figure 3). No hemorrhagic transformation was present on either study. She was ultimately transitioned to an oral anticoagulant. The patient was seen in the outpatient Vascular Neurology clinic ~1 month later, at which time she had no residual symptoms and had an NIH Stroke Score of 0. Additionally, due to severe proximal right ICA stenosis detected on an outpatient carotid artery duplex ultrasound, she ultimately underwent re-vascularization via proximal ICA angioplasty and stent placement. The patient continues to do well and is functionally independent.

## Discussion

A recent systematic review evaluating the effectiveness of tenecteplase vs. alteplase in patients with an AIS concluded that tenecteplase administration was associated with higher arterial recanalization rates than alteplase and that functional outcomes in patients were equivalent (Potla and Ganti, 2022). In addition, there did not appear to be a higher frequency of symptomatic intracerebral hemorrhage following treatment with tenecteplase. The efficacy and safety experiences with tenecteplase have, in part, triggered a rise in use of this thrombolytic drug in primary and comprehensive stroke centers within the U.S. when patients can be treated within the 0–4.5 time window from last known well. Also contributing to increased use of tenecteplase is its single bolus mode of administration, specifically due to its longer half-life. The single bolus augments quicker workflow in comparison with the 1-h infusion time associated with alteplase, particularly when patients must be transported from outlying emergency departments to stroke centers and when patients with large vessel occlusion are moved from an emergency department to an interventional suite. The EXTEND study demonstrated that alteplase therapy, when administered to patients with a favorable perfusion imaging profile between 4.5 and 9 h after stroke onset or on awakening with stroke (if within 9 h from the midpoint of sleep), resulted in a higher percentage of patients with no or minor neurologic deficits than the use of placebo (Ma et al., 2019). Data concerning the efficacy and safety of tenecteplase in this time window is lacking, thus alteplase continues to be the drug of choice when patients meet treatment and imaging criteria between 4.5 and 9 h for intravenous thrombolysis.

Due to the considerable frequency of heparin infusion use in clinical conditions (e.g., atrial fibrillation and hypercoaguable states) and interventional procedures that are associated with a risk of AIS, neurologists are not infrequently consulted to emergently assess patients who are receiving therapeutic dosing of heparin and are also experiencing symptoms of stroke. In addition, neurologists may encounter patients with AIS who have heparin induced thrombocytopenia and stroke symptoms. Approximately 0.5–1% of those exposed to unfractionated heparin can develop heparin induced thrombocytopenia (Arepally and Padmanabhan, 2020). This immune driven reaction can precipitate ischemic strokes via arterial, venous, or cardioembolic etiologies as it can induce a pro-thrombotic state (El Husseini and Morgenlander, 2011). Consequently, it is important that neurologists are aware of the ability to rapidly reverse the anticoagulant effects of heparin via protamine sulfate. This positively charged alkaline protein molecule binds to negatively charged heparin molecules and ultimately results in the formation of a neutral salt. Protamine sulfate has a rapid onset of action by effectively neutralizing unfractionated heparin's anticoagulant properties in < 5 min (Applefield and Krishnan, 2020). The dosing for heparin reversal is generally 1 mg of protamine sulfate for every 100 units of heparin as a slow intravenous push over 10 min, with a maximum dosage of 50 mg (Applefield and Krishnan, 2020).

In the cardiology literature, elevated PTT values in patients being treated with intravenous heparin have been associated with higher rates of intracerebral hemorrhage following fibrinolysis (Demaerschalk et al., 2016). There is, however, scarce data regarding a true cut point PTT above which alteplase administration is unsafe in patients with AIS. In 2016, an American Stroke Association Scientific Statement included a comment that the safety and efficacy of intravenous alteplase for acute stroke patients with an activated PTT >40 was unknown and thus not recommended (Demaerschalk et al., 2016). This recommendation was incorporated into the 2019 American Stroke Association Guidelines for the Early Management of Acute Ischemic Stroke (Powers et al., 2019). Protamine sulfate's rapid time of heparin neutralization, however, suggests that re-checking an activated PTT prior to administering thrombolytics may not be required, especially given the time sensitivity for thrombolysis in AIS (Kneer et al., 2023).

In a case series and literature review encompassing 11 patients who received alteplase after heparin reversal with protamine sulfate, two patients had small petechial hemorrhagic infarctions, but no symptomatic intracranial hemorrhages occurred (Ranasinghe et al., 2019). Although the number of cases in this report is small, the safety data is reassuring and can be incorporated into decision making among admitted patients who suffer an AIS while receiving heparin for medical issues such as myocardial infarction, atrial fibrillation, or deep venous thrombosis. With the ongoing transition from alteplase to tenecteplase as the preferred thrombolytic drug within U.S. stroke centers, it will be important to determine if the apparent safety of alteplase use following heparin reversal is also applicable to tenecteplase. Our case supports this supposition, but further reports that incorporating more cases will be necessary before a firm conclusion concerning the safety of this approach can be made.

## Data availability statement

The raw data supporting the conclusions of this article will be made available by the authors, without undue reservation.

## Ethics statement

Written informed consent was obtained from the individual(s) for the publication of any potentially identifiable images or data included in this article.

## Author contributions

MD: Writing—original draft, Writing—review & editing, Conceptualization, Data curation, Formal analysis, Funding acquisition, Investigation, Methodology, Project administration, Resources, Software, Supervision, Validation, Visualization. AF: Conceptualization, Data curation, Formal analysis, Funding acquisition, Investigation, Methodology, Project administration, Resources, Software, Supervision, Validation, Visualization, Writing—review & editing. KA: Conceptualization, Data curation, Formal analysis, Funding acquisition, Investigation, Methodology, Project administration, Resources, Software, Supervision, Validation, Visualization, Writing—review & editing. ME: Conceptualization, Data curation, Formal analysis, Funding acquisition, Investigation, Methodology, Project administration, Resources, Software, Supervision, Validation, Visualization, Writing—review & editing. SS: Conceptualization, Data curation, Formal analysis, Funding acquisition, Investigation, Methodology, Project administration, Resources, Software, Supervision, Validation, Visualization, Writing—review & editing.
